# *Mycobacterium avium* subsp. *paratuberculosis* Infected Cows Reveal Divergent Immune Response in Bovine Peripheral Blood Derived Lymphocyte Proteome

**DOI:** 10.3390/metabo12100924

**Published:** 2022-09-29

**Authors:** Lucia Korbonits, Kristina J. H. Kleinwort, Barbara Amann, Andrea Didier, Erwin Märtlbauer, Stefanie M. Hauck, Cornelia A. Deeg

**Affiliations:** 1 Faculty of Veterinary Sciences, LMU Munich, D-82152 Martinsried, Germany; 2 Faculty of Veterinary Sciences, LMU Munich, D-85764 Oberschleißheim, Germany; 3 Metabolomics and Proteomics Core, Helmholtz Zentrum München, German Research Center for Environmental Health, D-80939 Munich, Germany

**Keywords:** paratuberculosis, mycobacteria, host-pathogen response, immune system, quantitative label-free liquid chromatography tandem mass spectrometry, bovine

## Abstract

Bovine paratuberculosis is a serious chronic disease of the gastrointestinal tract that causes economic losses and dramatically affects animal health in livestock. The underlying infectious agent, *Mycobacterium avium* subspecies *paratuberculosis* (MAP), cannot reliably be detected by standard diagnostic tests due to the long asymptomatic disease stage. The aim of this study was to detect proteomic changes in peripheral blood mononuclear cells (PBMC) from cows of the same herd with different MAP infection status after co-incubation with viable MAP in vitro using label-free LC-MS/MS. In our proteomic discovery experiment, we detected 2631 differentially regulated proteins between cows with negative MAP infection status (so-called MAP-resistant cows) and cows with positive MAP infection status (so-called persistently MAP-infected cows). In MAP-resistant cows, we detected enriched immune-related signaling pathways for TLR2 and MHC class II component proteins, among others, indicating a successful defensive immune response of the cows to MAP. In contrast, persistently MAP-infected cows were not directly enriched in immune-related signaling pathways associated with ITGA2B and KCNMA1, among others. The introduction of these distinct immune responses contributes to a better understanding of the bovine immune response and mechanisms of susceptibility to MAP.

## 1. Introduction

*Mycobacterium avium* subspecies *paratuberculosis* (MAP) is the clinically and economically important agent of bovine paratuberculosis [1]. Commonly known as Johne’s disease, it is a debilitating chronic disease of the gastrointestinal tract that occurs worldwide in domestic and wild ruminants [2,3]. Because of its high tenacity, MAP is able to survive for about a year in soil and fresh water, which poses a risk because a single infected animal can threaten the health of the entire herd [4].

Fecal–oral transmission of MAP occurs primarily through ingestion of MAP-contaminated feces, colostrum, or milk from infected animals. Infection usually occurs in the first few months of life when older animals excrete the bacterium in their feces but remains subclinical until adulthood [5,6]. In clinically infected cattle it causes weight loss, diarrhea, and reduced milk yields, and therefore causes major economic losses for the farmer [3,7]. After infection, ruminants usually go through a long, asymptomatic subclinical phase during which MAP cannot reliably be detected by standard diagnostic tests [8,9]. These infected but unrecognized animals are the major spreaders of the disease in livestock and remain difficult to identify because available diagnostic tests are not sensitive enough to detect them [10].

The use of commercial inactivated vaccines against Johne’s disease in cattle is still limited in most countries and has not eradicated the disease where it is widespread [11]. In addition, vaccination interferes with tests to detect *Mycobacterium bovis* [12]. Therefore, current management strategies to control Johne’s disease aim to limit calf exposure to MAP by avoiding contact with both, adult cattle and their feces and by using aggressive testing and eradication practices [13,14]. However, forced culling strategies have generally failed to reduce MAP prevalence in livestock [13]. Voluntary sanitation and management practices have been used to reduce and prevent MAP transmission to susceptible cows [10]. However, MAP eradication programs based solely on hygiene management are not very promising [15]. In addition, MAP prevalence at the herd level did not decrease with farm participation in a Johne’s disease control program that included measurement of herd MAP infection status, risk assessment, and an individualized management plan [16]. We have previously shown that there are at least two different immune phenotypes in cattle in Germany [17]. Therefore, the aim of our study was to investigate whether the difference between MAP-resistant and persistently MAP-infected cows is due to differences in the anti-MAP immune response. Understanding differences in bovine immune responses to MAP could improve selection for natural resistance to MAP through breeding and complement existing MAP eradication programs. On farms where natural MAP infections are detected, there are always differences in the susceptibility of some cattle to the infection. In a previous study, we showed that co-incubation of MAP and peripheral blood mononuclear cells (PBMC) from cows from a MAP-free farm with different immunophenotypes responded to co-incubation with MAP with increased abundance of proteins that can promote MAP infection and persistence [18], while a classical IL-12-driven immune response was observed in control cows [8]. On farms with established MAP occurrence, some cows were more susceptible to MAP infection than others [10]. To gain deeper insights into this differential immune response of cows to MAP, our study tested PBMCs from cows with different natural MAP infection status—from animals in the infected herd that were confirmed MAP-negative (MAP-resistant cows) and from cows with confirmed positive MAP status (persistently MAP-infected cows). 

Because we were interested in an in-depth characterization of possible different immune responses, we used differential proteomic analysis to investigate the changes in the proteome between these two groups. The aim of this study was to investigate whether these cattle respond functionally differently to a 48-h infection with MAP in vitro and to characterize the different immune responses accordingly in more detail to obtain information on the role of the host immune response in combating MAP.

## 2. Materials and Methods

### 2.1. Selection of Animals and Detection of MAP Infection Status 

In this study, peripheral blood mononuclear cells (PBMC) from 14 cows were analyzed. To select animals for our study, we previously performed a very detailed characterization of the infection status of 31 animals on the dairy farm of interest. For generating the group “MAP-resistant cows”, we only included animals showing no positive results after bacterial cultivation of fecal and milk samples on commercial Herrold’s Egg Yolk Agars (HEYM agar, Becton Dickinson, Heidelberg, Germany) for 12 weeks, enzyme-linked immunosorbent assay (ELISA) with serum and milk samples (ID Screen Paratuberculosis Indirect, IDVet, Grabels, France; cattletype MAP Ab, Qiagen, Hilden, Germany), PCR with milk and fecal samples and Ziehl–Neelsen stainings from fecal samples. Assignment to the group “persistently MAP-infected cows” was done when animals showed positive results in the measuring methods mentioned above. After bacterial cultivation and ELISA, 17 animals could not be clearly assigned due to heterogeneous test results and were not considered for the analyses. Cows were at least 48 months and at the most six years of age at the time of initial sampling. Specifically, for mass spectrometry analysis of bovine PBMC samples, cells from four MAP-resistant (first four cows from negative group in Supplementary Table S5) and five persistently MAP-infected cows (first five cows from positive group in Supplementary Table S5) were examined. These animals all came from the same dairy farm with identical environmental conditions. To verify differential TLR2 and MHC class II expression by flow cytometry and CD41 and KCNMA1 expression by immunocytology, PBMC from two MAP-resistant (last two cows from negative group in Supplementary Table S5) and from two persistently MAP-infected cows (last two cows from positive group in Supplementary Table S5) from another dairy farm were examined. These four animals all came from the same dairy farm with identical environmental conditions. The MAP status of these cows was determined by bacterial culturing of fecal samples on HEYM agar for 12 weeks and ELISA with serum samples. The collection of bovine venous whole blood and the experimental protocols were approved by the Government of Upper Bavaria, Munich (approval no. ROB-55.2-2532.Vet_03-17-106). No experimental animals were used in this study. Permission was obtained from dairy farms to use blood samples from their animals for study purposes.

### 2.2. Preparation of PBMC and Co-Incubation of PBMC with Viable MAP In Vitro

Venous bovine whole blood was collected in tubes coated with sodium heparin (25,000 I.U.). Blood was diluted 1:2 in PBS (NaCl 136.9 mM, Na_2_HPO_4_ × 2H_2_O 8.1 mM, KH_2_PO_4_ 1.4 mM, and KCl 2.6 mM; pH 7.2) and isolation of PBMC was performed by density gradient centrifugation (room temperature, 500× *g*, 25 min, decelerate) using Pancoll separating solution (PanBiotech, Aidenbach, Germany). PBMC were recovered from the intermediate phase, washed twice in PBS, and used for in vitro co-incubation with viable MAP immediately. After resuspension in RPMI 1640 (PanBiotech) with 1% penicillin-streptomycin (PanBiotech), bovine PBMC (5 × 10^6^ cells) were co-incubated with viable MAP of strain DSM 11023 at a multiplicity of infection (MOI) of 4:1 at 37 °C and 5% CO_2_. MAP were obtained from the German Collection of Microorganisms and Cell Cultures (DSMZ, Braunschweig, Germany) and grown in Middlebrook 7H9 broth (VWR, Ismaning, Germany) supplemented with Middlebrook enrichment ADC medium (VWR, Ismaning, Germany), Mycobactin J (IDVet, Grabels, France), 0.002% glycerol, and 0.03% Tween 80. After 48 h, cells were washed twice with PBS and the supernatant was discarded before proteomic analysis. For flow cytometry and immunocytological analyses, after 48 h of incubation with viable MAP (strain DSM 11023, MOI of 4:1) in vitro, cells were washed twice with PBS, supernatants were discarded, and PBMC were processed immediately.

### 2.3. Sample Digestion for Differential Proteome Analysis

From each PBMC sample, 6 × 10^5^ cells were digested using a modified FASP protocol, as described [19]. Samples were separately lysed directly in 200 µL urea buffer (UA buffer, Sigma-Aldrich, Saint Louis, MO, USA) with the addition of 1 µL 1 M dithiothreitol (DTT), then shaken for 30 min at room temperature and diluted 1:2 with UA buffer. After addition of 10 µL of 300 mM iodoacetamide, samples were shaken for 30 min at room temperature in the dark. 2 µL of 1 M DTT was added to quench unreacted 2-iodoacetamide. 30 kDa cut-off centrifuge filters (Sartorius, Göttingen, Germany) were equilibrated with 100 µL UA buffer and centrifuged at 15,000× *g* for 10 min. Samples were subsequently transferred to the filters by centrifugation at 15,000× *g* and washed three times with 200 µL UA buffer at 15,500× *g* and three times with 100 µL 50 mM ammonium bicarbonate buffer (ABC buffer, Sigma-Aldrich, Saint Louis, MO, USA). After washing, proteins were subjected to proteolysis for 2 h at room temperature by adding 0.5 µg lysyl endopeptidase in 40 µL ABC buffer to the filter, followed by the addition of 1 µg trypsin and 10 µL ABC buffer and incubation at 37 °C overnight. Peptides were collected by centrifugation over the filter, and 20 µL of ABC buffer containing 5% acetonitrile was added to the filter. After a second centrifugation at 15,900× *g* for 20 min at room temperature, the eluates were combined and acidified with trifluoroacetic acid to give a pH of 2. 

### 2.4. Mass Spectrometric Analysis and Protein Identification

Peptide analysis of each PBMC sample was performed as previously described [17]. No technical replicates were used. Acidified eluted peptides were analyzed in data-dependent mode on a Q Exactive HF mass spectrometer (Thermo Fisher Scientific, Bremen, Germany) coupled on-line to an UItimate 3000 RSLC nano-HPLC (Dionex, Sunnyvale, CA, USA). Samples were automatically injected and loaded onto the Nanotrap column, eluted after 5 min, and separated from 2 to 40 percent ACN on the analytical column (75 µm inner diameter × 25 cm, Acclaim PepMap100 C18, 3 µm, 100 Å) by a 265-min gradient flow at a flow rate of 300 nL/minute. By using LTQ OrbitrapXL, peptides were analyzed with acquired MS spectra resolution at 60,000 in profile mode. The ten most intense peptide ions were chosen for fragment analysis in the linear ion trap if they were at least doubly charged and surpassed an intensity of at least 200 counts after the high-resolution prescan. The normalized collision energy for CID was set to a value of 35, and the resulting fragments were detected at normal resolution in the linear ion trap in centroid mode and dynamic exclusion was set to 60 s. 

The raw spectra were imported into Progenesis QI (version 2.5, Waters, Milford, MA, USA) software. The spectra were exported as Mascot Generic files and further processed with Mascot [20] (version 2.5.1, Matrix Science, Boston, MA, USA) with the search parameters in the Ensembl bovine database (version 93, number of coding genes: 21,880) as follows: 10 ppm peptide mass tolerance and 20 mmu fragment mass tolerance, one missed cleavage was allowed, carbamidomethylation was set as a fixed modification, methionine oxidation and asparagine or glutamine deamidation were allowed as variable modifications. Assignments of spectra to sequences is based on software algorithms and undergoes a quality cut-off. A Mascot-integrated Decoy database search resulted in an average false discovery of <1% when the search was performed with a Mascot percolator score cut-off of 13 and an appropriate significance threshold p.

Peptide assignments were imported into Progenesis QI software, and the abundances of all individual peptides assigned to each protein were summed up. The resulting normalized abundances of each protein were used to calculate fold-changes of protein ratios between conditions. Statistical analysis was performed on the log2-transformed normalized abundance values using Student’s *t* test. At *p* < 0.05, changes in protein expression between conditions were considered significant.

### 2.5. Data Processing 

For statistical analysis, transformed normalized frequencies were used to calculate Student’s *t* test. Proteins were considered significant if *p* ≤ 0.05 and were used for further analysis. No cutoff value was set for the ratio of persistently MAP-infected to MAP-resistant cows for proteins. Pathway enrichment analyses were performed using the open-source software ShinyGO (version 0.76, http://bioinformatics.sdstate.edu/go/, accessed on 8 July 2022) with the following settings: Search Species Cow, *p*-value cutoff (FDR) ≤ 0.05. The *p*-value for enrichment analysis was calculated using hypergeometric distribution followed by FDR correction. 

### 2.6. Flow Cytometric Analysis of PBMC 

PBMC from four cows, two MAP-resistant and two persistently MAP-infected cows, were stained in 96-well round-bottom plates with 2 × 10^5^ cells per well. All antibodies were incubated at 4 °C for 20 min. The bivalent CD282:FITC antibody (clone AbD12542, Bio-Rad AbD Serotec, Puchheim, Germany, 1:25) or the monoclonal mouse anti-horse MHC class II IgG1 antibody cross-reactive with bovine MHC class II (mAb clone CVS20, Bio-Rad AbD Serotec, Puchheim, Germany, 1:100) was incubated with the cells. After washing with PBS, the secondary antibody F(ab’)2 fragment goat against mouse IgG Alexa 488 (Dianova, Hamburg, Germany, 1:400) was added to the MHC class II-stained cells. Positive controls were mouse IgG1 antibodies against human CD79a cross-reactive with bovine CD79a (mAb clone HM57, Bio-Rad AbD Serotec, Puchheim, Germany, 1:100), against bovine CD4 (mAb clone CC30, Bio-Rad AbD Serotec, Puchheim, Germany, 1:200), against bovine CD3 (mAb clone MM1A, Thermo Fisher Scientific, Bremen, Germany, 1:100), IgG2a antibodies against human MHC class I, cross-reactive with bovine MHC class I (mAb clone W6/32, Bio-Rad AbD Serotec, Puchheim, Germany, neat) and against bovine CD8 (mAb clone CC63, Bio-Rad AbD Serotec, Puchheim, Germany, 1:100) and mouse IgG2b antibodies against bovine TCR1 (mAb clone GB21A, γδ T cells, Biomol, Hamburg, Germany, 1:100) were used. After washing with PBS, the secondary antibody F(ab’)2 fragment goat against mouse IgG Alexa 488 (Dianova, Hamburg, Germany, 1:400) was added to the stained cells. Cell viability was determined by staining with Viobility 400/452 Fixable Dye (Miltenyi Biotec, Bergisch Gladbach, Germany). Only viable cells were included in further analyses. Measurements were performed using a NovoCyte Quanteon flow cytometer (Agilent, Waldbronn, Germany) and results were analyzed using Flowlogic Software V7 (Miltenyi Biotec, Bergisch Gladbach, Germany).

### 2.7. Immunocytology and Quantification of Signal Intensities

PBMC from four cows, two MAP-resistant and two persistently MAP-infected cows, were stained in 96-well round bottom plates with 2 × 10^5^ cells per well. All antibodies were incubated at 4 °C for 20 min. Mouse anti-bovine CD41 (mAb clone CAPP2A, IgG2b, Biomol, Hamburg, Germany, 1:200) or rabbit anti-human KCNMA1 antibody cross-reacting with bovine KCNMA1 (polyclonal, Bio-Rad AbD Serotec, Puchheim, Germany, 1:100) were incubated with the cells. After washing with PBS, an F(ab’)2 fragment of a goat anti-mouse IgG Alexa 488 secondary antibody (Dianova, Hamburg, Germany, 1:400) or a highly cross-absorbing goat anti-rabbit IgG (H + L) Alexa Fluor 488 secondary antibody (Thermo Fisher Scientific, Bremen, Germany, 1:400) was added. Cells were fixed with 1% paraformaldehyde [21] and nuclei were counterstained with 4′,6-diamidino-2-phenylindole (DAPI, Thermo Fisher Scientific, Bremen, Germany). The fixed cells were transferred to slides, and sections were mounted on glass coverslips with non-fluorescent mounting medium (SERVA, Heidelberg, Germany). Fluorescence images were acquired with a Leica DMi8 microscope, and LASX software, version 3.4.2 (both Leica Microsystems, Wetzlar, Germany) was used for image processing. Mean gray factor was measured in representative areas for quantification, and results were compared between MAP-resistant and persistently MAP-infected cows. The Mann–Whitney U test was used to analyze differences in staining intensity because the Kolmogorov–Smirnov test did not reveal a normal distribution of the data using the mean gray factor. The Gaussian distribution was determined using the Kolmogorov–Smirnov test. Results were considered significant at *p* ≤ 0.05. Significances were indicated by asterisks with * *p* ≤ 0.05, ** *p* ≤ 0.01, and *** *p* ≤ 0.001. Data were processed, analyzed, and visualized using GraphPad Prism version 5.04 (GraphPad Software, San Diego, CA, USA).

## 3. Results

### 3.1. The Proteome of Bovine Peripheral Blood Lymphocytes Consisted of 2631 Proteins and Showed Significant Differences between MAP-Resistant and Persistently MAP-Infected Cows after 48 h of Co-Incubation with MAP In Vitro 

Using LC-MS/MS analysis, we identified the proteome of bovine peripheral blood mononuclear cells (PBMC), which included 2631 proteins. The high number of identified proteins reflects the analytical depth due to standard sample preparation and state-of-the-art analytical methods. After 48 h of co-incubation with *Mycobacterium avium* subsp. *paratuberculosis* (MAP) in vitro, we found significant (*p* ≤ 0.05) changes in lymphocyte protein abundances between the two groups. After co-incubation with MAP in vitro, the proteomes of PBMC from MAP-resistant and persistently MAP-infected cows showed significant differences. Fifty-one proteins of the lymphocyte proteome from MAP-resistant cows were more abundant, whereas 102 proteins showed increased abundance in lymphocytes from persistently MAP-infected cows. 

### 3.2. Analyses of Enriched Signaling Pathways Revealed Functional Differences between Lymphocytes from MAP-Resistant and Persistently MAP-Infected Cows after Co-Incubation with MAP In Vitro

**Figure 1:**
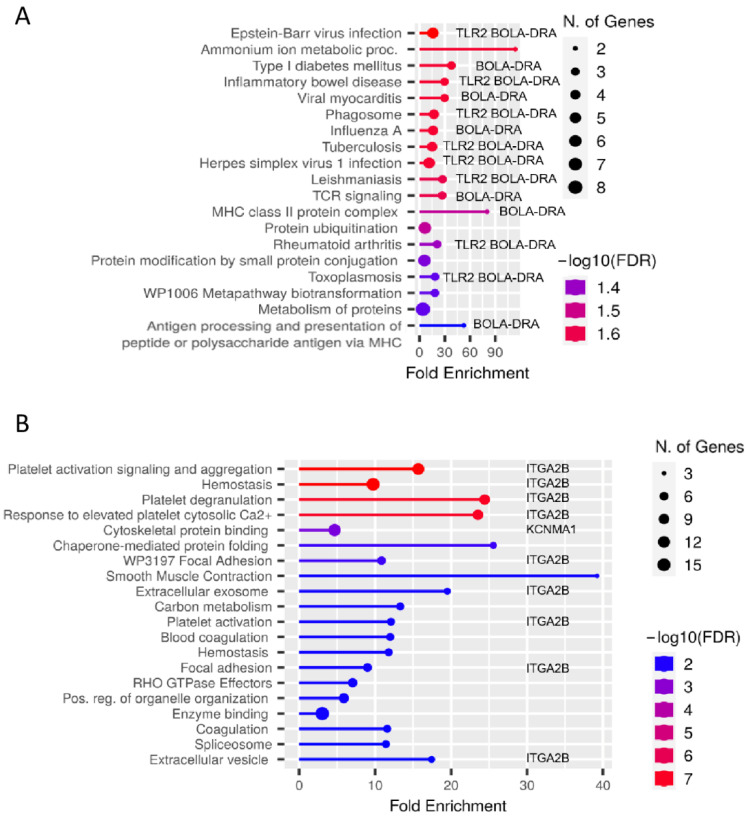
Analysis of functional enrichment of proteins with different abundance (*p* ≤ 0.05) in PBMC of (**A**) MAP-resistant and (**B**) persistently MAP-infected cows after 48 h incubation with MAP in vitro. Functional enrichment shows the 20 most significant categories of all available gene sets. Hierarchical clustering was performed using ShinyGO. The y-axis lists the assigned pathways in order of FDR values for the enrichment of each pathway. The x-axis shows the values in order of FDR enrichment for related paths. The color map shows the enrichment of the FDR for each path. The size of the dots corresponds to the number of genes associated with each pathway. The corresponding pathway enrichment data for MAP-resistant and persistently MAP-infected cows are shown in more detail in Tables S1 and S2. (**A**) Bos taurus toll-like receptor 2 (TLR2) and Bos taurus major histocompatibility complex, class II, DR alpha (BOLA-DRA, MHC class II) were associated with several signaling pathways enriched in lysates from MAP-resistant cows after 48-h incubation with MAP in vitro, whereas (**B**) Bos taurus integrin, alpha 2b (ITGA2B, CD41) and Bos taurus potassium large conductance calcium-activated channel, subfamily M, alpha member 1 (KCNMA1) were associated with enriched signaling pathways in PBMC from persistently MAP-infected cows after 48-h incubation with MAP in vitro.

Our hypothesis-generating approach aimed to clarify the functional effects of the different proteome of cows from the same herd with different MAP status after contact with MAP in vitro. To this end, we used all proteins with significantly (*p* ≤ 0.05) different abundance between groups to perform pathway enrichment analysis. Interestingly, among the proteins significantly enriched in MAP-resistant individuals, pathways such as “Epstein–Barr virus infection”, “Ammonium metabolic processes”, “Diabetes mellitus type I”, and “Inflammatory bowel disease” were enriched due to the higher abundance of “Toll-like receptor 2” and “Major histocompatibility complex, class II, DR alpha” proteins (Figure 1A). 

In contrast, the enriched pathways in persistently MAP-infected cows were not directly immune-related pathways labeled “platelet activation signaling and aggregation”, “hemostasis”, “platelet degranulation”, and “cytoskeletal protein binding” (Figure 1B). In association with these enriched signaling pathways, integrin alpha 2b (ITGA2B, CD41) and potassium large conductance calcium-activated channel, subfamily M, alpha member 1 (KCNMA1), among others, were significantly enriched in persistently MAP-infected cows after in vitro co-incubation with MAP (Figure 1B). 

### 3.3. In Vitro Co-Incubation with MAP for 48 h Increased the Abundances of TLR2, BOLA-DRB3, and BOLA-DRA in MAP-Resistant Cows

**Figure 2:**
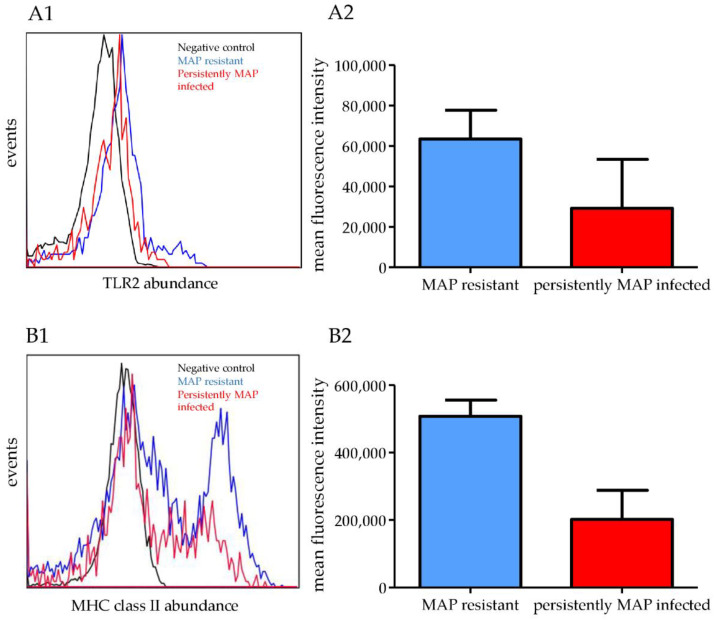
Higher abundance of TLR2 and MHC class II in PBMC of MAP-resistant compared with persistently MAP-infected cows. (**A1**) Higher abundance of TLR2 in PBMC from MAP-resistant compared with persistently MAP-infected cows as measured by flow cytometry analysis with representative histograms. (**A2**) Higher mean fluorescence intensity of TLR2 positive PBMC from MAP-resistant compared to persistently MAP-infected cows. Mean fluorescence intensity from flow cytometry ± SD (**B1**) Higher MHC class II abundance in PBMC from MAP-resistant compared with persistently MAP-infected cows as measured by flow cytometry analysis with representative histograms. (**B2**) Higher mean fluorescence intensity of MHC class II positive PBMC from MAP-resistant compared to persistently MAP-infected cows. Mean fluorescence intensity from flow cytometry ± SD.

In our analysis of differential proteomic changes in MAP-resistant cows, a term we use to describe cows that are less susceptible to infection with MAP, we observed a significant increase in the expression of TLR2, BoLA-DRB3, and BoLA-DRA in PBMC after co-incubation with MAP in vitro. Cells from MAP-resistant cows responded to MAP co-incubation with a 2.5-fold higher expression of TLR2 (*p* = 0.012) compared with cells from persistently MAP-infected cows. Therefore, we examined the increase in expression of TLR2 in lymphocytes from MAP-resistant cows by flow cytometry (Figure 2(A1,A2)). Interestingly, after co-incubation with MAP, MAP-resistant cows expressed 2.5-fold more BoLA-DRB3 (*p* = 0.017) than cells from persistently MAP-infected cows. In addition, BoLA-DRA (*p* = 0.044) was enriched 1.4-fold more in cells from MAP-resistant than in cells from persistently MAP-infected cows. The data for MAP-resistant cows are shown in Supplemental Table S3.

We detected higher expression and higher mean fluorescence intensity of TLR2 on the cell surface of lymphocytes from MAP-resistant cows (Figure 2(A1,A2)). Because BoLA-DRB3 and BoLA-DRA are both proteins associated with class II of the major histocompatibility complex, we examined MHC class II expression on PBMC from MAP-resistant and persistently MAP-infected cows. There was a marked increase in MHC class II expression on the cell surface of lymphocytes from MAP-resistant cows (Figure 2(B1,B2)). Differential expression of TLR2 and MHC class II on the cell surface of PBMC from MAP-resistant and persistently MAP-infected cows was demonstrated by flow cytometry analysis with additional animals from another dairy farm (Figure 2).

### 3.4. Significantly Higher Expression of ITGA2B and KCNMA1 in Persistently MAP-Infected Cows after Co-Incubation with MAP In Vitro

In contrast to the differences observed in the PBMC proteomes of MAP-resistant cows, the expression of ITGA2B and KCNMA1 was significantly higher in the lymphocytes of persistently MAP-infected cows after co-incubation with MAP. Persistently MAP-infected cows expressed 2.2-fold more ITGA2B (*p* = 0.047) than cells from MAP-resistant cows after co-incubation with MAP. In addition, cells from persistently MAP-infected cows responded to co-incubation with MAP with 15.2-fold higher expression of KCNMA1 (*p* = 0.004) compared with cells from MAP-resistant cows. The data for persistently MAP-infected cows are shown in Supplemental Table S4.

**Figure 3:**
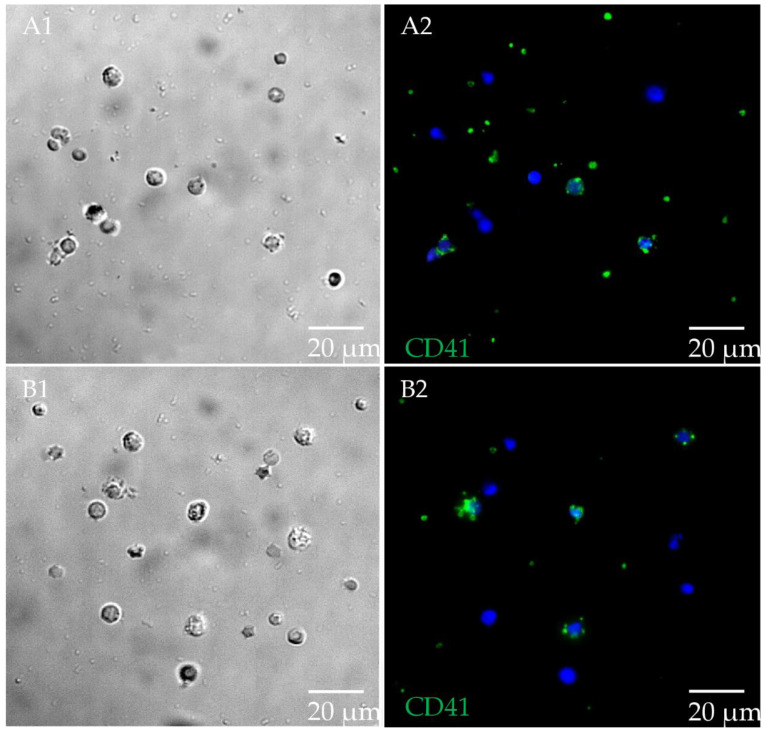
CD41 on PBMC from MAP-resistant and persistently MAP-infected cows. (**A1**) Differential interference contrast (DIC) imaging of PBMC from MAP-resistant cows. (**A2**) CD41 (in green) in PBMC from MAP-resistant cows (nuclei stained with 4′,6-diamidino-2-phenylindole (DAPI)). CD41 expression identified on platelets and PBMC from MAP-resistant cows. (**B1**) DIC of PBMC from persistently MAP-infected cows. (**B2**) CD41 (in green) in persistently MAP-infected cows. CD41 expression on platelets and PBMC of persistently MAP-infected cows (all representative images). Image sizes are annotated by scale bar in lower right corner of respective image.

**Figure 4:**
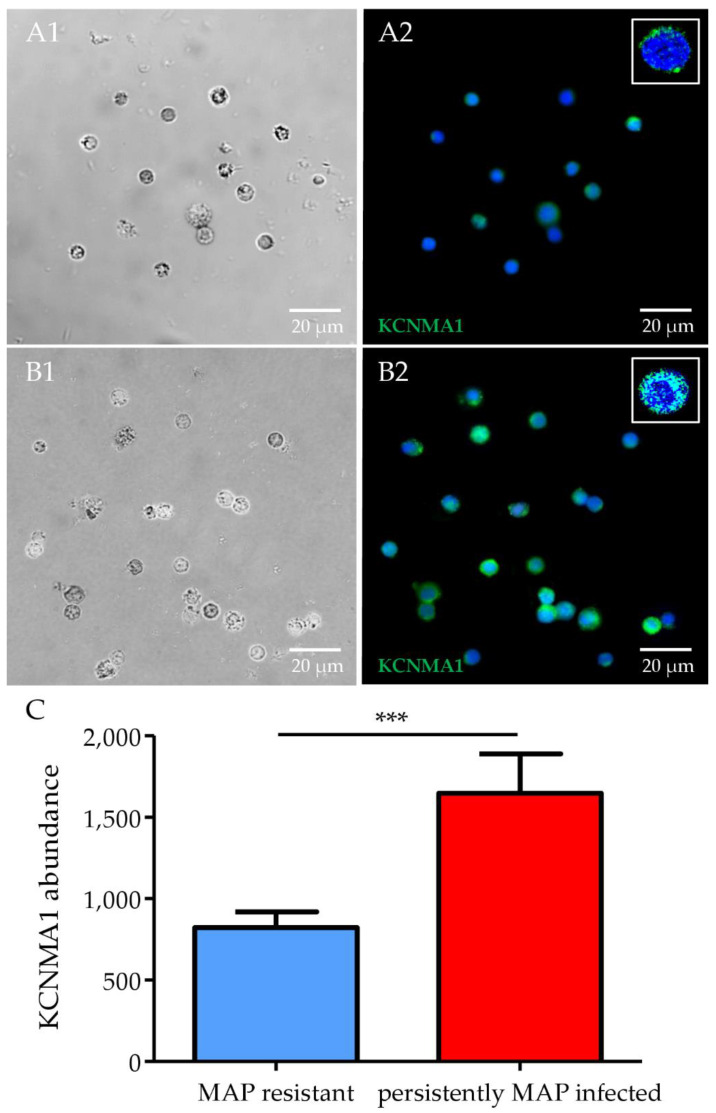
Higher abundance of KCNMA1 in PBMC of MAP-resistant compared with persistently MAP-infected cows. (**A1**) Differential interference contrast (DIC) imaging of PBMC from MAP-resistant cows. (**A2**) KCNMA1 (in green) in PBMC of MAP-resistant cows (nuclei stained with 4’,6-diamidino-2-phenylindole (DAPI)). (**B1**) DIC of cells from persistently MAP-infected cows. (**B2**) More KCNMA1 (in green) in persistently MAP-infected cows (all representative images). (**C**) Significantly higher KCNMA1 abundance in persistently MAP-infected cows than in MAP-resistant ones (quantified mean gray abundance from immunocytology ± SD, *** *p* ≤ 0.001). Image sizes are annotated by scale bar in lower right corner of respective image.

We detected cell expression of CD41, the product of the ITGA2B gene, on bovine PBMC. Based on cell expression of CD41 on bovine platelets and cell adhesion of platelets to PBMC, we could not quantify differential expression of CD41 on bovine PBMC from MAP-resistant compared with persistently MAP-infected cows. Because we could not technically detect CD41 expression in flow cytometry, we show CD41 expression of MAP-resistant compared with persistently MAP-infected cows measured by immunocytology. (Figure 3). We were able to detect the expression of KCNMA1 in PBMC from cattle. Moreover, we detected significantly (*p* ≤ 0.001) higher KCNMA1 abundance on the cell surface in PBMC from persistently MAP-infected cows compared to MAP-resistant ones (Figure 4). 

## 4. Discussion

The importance of JD is undisputed for animal welfare and economic reasons [22]. PBMCs are an essential component of the immune system of cattle and contribute to the defense against bacteria through activation, immune response, and inflammatory response [23].

TLR2, a pattern recognition factor, is present on the surface of bovine cells and is highly expressed by peripheral blood monocytes; it plays an important role in eliciting immune responses to mycobacteria [24,25]. In our study, we demonstrated higher abundance of TLR2 in the proteome and on the cell surface of PBMC from MAP-resistant compared to persistently MAP-infected cows from two different dairy farms (Figure 2). In an in vivo infection study, TLR2 was markedly downregulated in response to MAP in PBMC from cattle experimentally inoculated with MAP, indicating a role for TLR2 in the pathogenesis of paratuberculosis [26]. The lower abundance of TLR2 in PBMC from cows persistently infected with MAP is due to either downregulation of TLR2 by MAP or a differential host immune response. An important link between protection against mycobacteria and TLR2 regulation was demonstrated in TLR2-/- mice, which, compared with wild-type mice, exhibited higher susceptibility to *M. tuberculosis* infection, showed decreased bacterial clearance and defective granulomatous response, and developed chronic pneumonia [27]. Therefore, downregulation of TLR2 in PBMCs from cows persistently infected with MAP suggests an unsuccessful immune response that is likely involved in the development of persistence of MAP in these cows. In human THP-1 cells, a monocytic leukemia cell line, it has been suggested that a TLR2-expressing cluster of classically differentiated macrophages exhibits the best defense response against MAP infection by increasing the expression of proinflammatory cytokines and chemokines such as IL1B, CCL4, CCL3, and CCL20 [28]. We hypothesized that MAP-resistant individuals choose a more successful immune response to combat MAP by upregulating TLR2.

MHC class II is a protein complex involved in the initiation of an inflammatory response by antigen presentation leading to macrophage activation, and is expressed on the surface of bovine PBMCs [29]. The more abundant proteins in PBMC of MAP-resistant cows were assigned to the enriched pathways “MHC class II protein complex” “antigen processing”, and “presentation of peptide or polysaccharide antigens via MHC” (Figure 1A). Proteins belonging to the MHC complex (bovine leukocyte antigen: BoLA), respectively, BoLA-DRA and BoLA-DRB3, showed induction of antigen presentation on PBMC of MAP-resistant cows after contact with MAP in vitro. The BoLA-DRB3 allele, which encodes the β-chain in the class II antigen complex, is the only gene described as functional among the three DRB loci in cattle [30]. In summary, we found that MHC class II abundance was higher in MAP-resistant cows than in persistently MAP-infected cows, possibly indicating that upregulation of MHC class II in PBMC is required as an appropriate defense mechanism against MAP infection. In contrast, MHC class II was actually decreased in persistently MAP-infected cows. We cannot explain this response to in vitro MAP infection at the moment, but it probably suggests that MAP causes the decreased MHC class II expression. In an in vitro infection study, MHC class II proteins were decreased in bovine macrophages after infection with MAP, whereas the decrease in MHC class II was not detected or was much less after infection with nonvirulent *M. avium* subsp. avium, illustrating a strategy of MAP to delay presentation and subsequent recognition by the adaptive immune system that may allow mycobacteria to persist in hosts [31]. In mice, a recombinant Bacillus Calmette–Guérin (BCG) vaccine against *M. tuberculosis* was used to ameliorate defects of BCG such as phagosome maturation, autophagy, and reduced MHC class II expression [32]. Recombinant BCG was found to induce robust MHC class II-dependent antigen presentation on CD4 T cells in vitro, activating TLR2 and thus leading to a better protection against tuberculosis in mice [32]. Interestingly, we also detected “phagosome” as one of the upregulated pathways associated with upregulated proteins in the PBMC proteome of MAP-resistant cows, which in turn is related to TLR2 and BoLA-DRA (Figure 1A). In nonpathogenic mycobacteria, phagosomes take up mycobacteria that fuse with lysosomes during maturation [32]. In contrast, *M. tuberculosis* inhibits phagosomal maturation and reduces MHC class II antigen processing, allowing survival in macrophages and providing a strategy for mycobacteria to evade immune surveillance [33]. Therefore, phagosomes from MAP-resistant cows could ingest and fuse with lysosomes during maturation, promoting adequate defense against mycobacteria. Investigation of possible defects in phagosome activity of persistently infected cows is planned to clarify the defects in immune response of persistently MAP-infected cows. This study demonstrates the importance of MHC class II and TLR2 in the defense against mycobacteria, as the abundance of TLR2 and MHC class II was higher in MAP-resistant cows than in persistently infected cows. 

In our in vitro MAP co-incubation study with bovine PBMC from persistently MAP-infected cows compared to MAP-resistant ones, we observed a higher abundance of ITGA2B in PBMC from persistently MAP-infected cows. ITGA2B, also known as antigen CD41, is commonly known as a platelet surface marker [34]. To our knowledge, we are the first to describe a possible association between higher susceptibility to infection with MAP and higher expression of ITGA2B on bovine PBMC. ITGA2B is not only present in platelet morphology but is also expressed on PBMC (Figure 3). Adhesion of platelets to PBMC in our experiments prevented clear differentiation of PBMC from platelets in our assay (Figure 3); therefore, we could not verify the result of differential proteomic analyses in this study. To elucidate whether ITGA2B could have a clinical use in MAP diagnostics, further studies should be applied with animals in different stages of infection. Moreover, as we only included animals with clear infection status to our study, testing of PBMC from animals which could not clearly be differentiated into negative or positive by current diagnostic tests should be analyzed for ITGA2B expression. Differential expression of ITGA2B was recently demonstrated in a proteomic study of bovine monocytes [35]. This study aimed to investigate the role of bovine monocytes during the non-cytopathic and cytopathic biotypes of bovine viral diarrhea virus (BVDV) in cows, demonstrating upregulation of ITGA2B in bovine monocytes during cytopathic BVDV infection [35]. Clinically, non-cytopathic BVDV infection can be followed by acute infection with cytopathic BVDV, which causes the fatal disease “mucosal disease” [35]. In a mouse lung model of tuberculosis, CD41 was highly expressed in lung areas with severe TB pathology with inflammation and tissue remodeling and was associated not only with nucleated cells but also with some nucleated cells, which was attributed to either staining of the marker on nucleated cells, phagocytosis of platelets, or adhesion of platelets to nucleated cells [36]. Therefore, the finding of upregulated ITGA2B on PBMC from persistently MAP-infected cows is, in our opinion, very interesting, and the underlying mechanisms deserve further investigation in future studies.

In addition, we detected higher expression of the calcium-activated potassium ion channel KCNMA1 in the PBMC proteome of persistently MAP-infected cows. We detected KCNMA1 on bovine PBMC for the first time and elucidated a significant (ratio: 15.2, *p* ≤ 0.001) KCNMA1 abundance in persistently MAP-infected cows after contact with viable MAP in vitro. To date, KCNMA1 has not been reported to be associated with the immune response to mycobacteria. Gene expression analysis of endometrial tissue of gravid heifers revealed a lower abundance of KCNMA1 compared with non-gravid heifers and has been discussed as an early response marker for maternal recognition of pregnancy [37]. The versatility of KCNMA1 due to multiple Ca^2+^ perception sites explains the presence of these channels in different human cell types, in which Ca^2+^ concentrations can vary widely [38]. In a recent transcriptome study of porcine small intestinal epithelial cells obtained from tissue samples of large white piglets, upregulation of KCNMA1 was observed in pigs with a phenotype susceptible to enterotoxigenic *Escherichia coli* (ETEC) F4 compared with naturally resistant pigs [39]. Pigs susceptible to ETEC express higher levels of KCNMA1 in epithelial cells of the small intestine than pigs with a naturally resistant phenotype, which is due to a functional ETEC F4 receptor [39]. Another transcriptome study on human PBMC described that KCNMA1 is upregulated in PBMC from patients with type 1 diabetes mellitus compared with a healthy control group [40]. It was hypothesized that the upregulation of KCNMA1 triggers an inflammatory response in PBMC from patients with type 1 diabetes [40]. Although these transcriptome data suggest a role of KCNMA1 in human and porcine disease susceptibility, transfer of these insights to our proteome-based findings needs to be interpreted with care due to transcriptome–proteome correlation discrepancy [41,42]. Nevertheless, the selective distinct upregulation of KCNMA1 in bovine PBMC is a highly interesting finding, suggesting an important role of this molecule in unsuccessful MAP defense. As suggested for ITGA2B, PBMC from animals which could not clearly be differentiated to the groups MAP-resistant or persistently MAP-infected cows by current diagnostic tests should be tested for KCNMA1 expression. Therefore, we aim to elucidate clinical use of KCNMA1 in MAP diagnostics in future studies. Because the physiological function of KCNMA1 on bovine PBMC is still unknown, further fundamental work is needed here to better understand its role in immune defense. To expand the insight given through the proteome data presented in this study, we will perform additional transcriptome-based experiments in the future.

According to our study, MHC class II and TLR2 are important proteins for successful immune defense against MAP in cows. In contrast, we can demonstrate for the first time a significant increase of ITGA2B and KCNMA1 in the immune response of cows with persistent MAP infection. The underlying mechanisms are currently unknown and should be further investigated.

## 5. Conclusions

We found significant differences in PBMC immune responses of MAP-resistant compared with persistently MAP-infected cows after contact with MAP in vitro. We hypothesize that the higher abundance of MHC class II complex proteins and TLR2 are important mechanisms for successful immune defense against MAP in cows. In contrast, the immune response of cows with persistent MAP infection showed significantly higher abundance of ITGA2B and KCNMA1 for the first time. The underlying mechanisms are currently unknown and should be further investigated.

## Data Availability

The raw mass spectrometry proteomics data have been deposited to the ProteomeXchange Consortium via the PRIDE [43] partner repository (https://www.ebi.ac.uk/pride/archive, accessed on 21 September 2022) with the dataset identifier PXD036881.

## Supplementary Materials

The following supporting information can be downloaded at: https://www.mdpi.com/article/10.3390/metabo12100924/s1, Supplemental Table S1: pathway enrichment data for MAP-resistant, Supplemental Table S2: pathway enrichment data for persistently MAP-infected cows, Supplemental Table S3: Selection of proteins with different abundance in MAP resistant cows after 48-h incubation with MAP in vitro, Supplemental Table S4: Selection of proteins with different abundance in persistently MAP-infected cows after 48-h incubation with MAP in vitro, Supplemental Table S5: Age distribution of cows used in study.
