# Supplementary material for: Mycobacterium avium subsp. paratuberculosis Infected Cows Reveal Divergent Immune Response in Bovine Peripheral Blood Derived Lymphocyte Proteome
Source: Metabolites. 2022 Sep 29;12(10):924. doi: 10.3390/metabo12100924 (PMC9608910; doi:10.3390/metabo12100924)
Supplement: Supplementary file 1 [file metabolites-12-00924-s001.zip › Supplementary Table S3_Korbonits et al.pdf]

Table S3. Selection of proteins with different abundance in MAP resistant cows after 48-h incubation with MAP *in vitro*. (a) Protein names as listed in Ensembl Protein Database v.93 (http://www.ensembl.org). (b) Gene names of Bos taurus (cow) (c) Protein accession numbers as listed in Ensembl database for cows v.93. (d) Fold change of differences in protein abundance in MAP-resistant compared to persistently MAP-infected cows (Up in MAP-resistant). Name of gene as listed in UniProtKB. (e) Statistical significance of differences in protein abundance determined by Student's t-test. Up-regulated genes in MAP-resistant: Bos taurus Toll-like receptor 2 (TLR2), Bos taurus major histocompatibility complex, class II, DRB3 (BOLA-DRB3), and Bos taurus major histocompatibility complex, class II, DR alpha (BOLA-DRA).

(a) **Protein name**	(b) **Gene name**	(c) **Accession number**	(d) **Ratio**	(e) **p-value**
**Upward in MAP Resistant**				
Bos taurus Toll-like receptor 2	*TLR2*	ENSBTAP000010530	2.5	0.012
Bos taurus major histocompatibility complex, class II, DRB3.	*BOLA-DRB3*	ENSBTAP000018484	2.5	0.017
Bos taurus major histocompatibility complex, class II, DR alpha	*BOLA-DRA*	ENSBTAP00000014072	1.4	0.044
