# Supplementary material for: Mycobacterium avium subsp. paratuberculosis Infected Cows Reveal Divergent Immune Response in Bovine Peripheral Blood Derived Lymphocyte Proteome
Source: Metabolites. 2022 Sep 29;12(10):924. doi: 10.3390/metabo12100924 (PMC9608910; doi:10.3390/metabo12100924)
Supplement: Supplementary file 1 [file metabolites-12-00924-s001.zip › Supplementary Table S4_Korbonits et al.pdf]

Table S4. Selection of proteins with different abundance in persistently MAP-infected cows after 48-h incubation with MAP *in vitro*. (a) Protein names as listed in Ensembl Protein Database v.93 (http://www.ensembl.org). (b) Gene names of Bos taurus (cow) (c) Protein accession numbers as listed in Ensembl database for cows v.93. (d) Fold change in protein abundance differences in persistently MAP-infected cows compared to MAP-resistant (Up in persistently MAP-infected cows). Name of gene as listed in UniProtKB. (e) Statistical significance of differences in protein abundance determined by Student's t-test. Genes upregulated in persistently MAP-infected cows: Bos taurus potassium large conductance calcium-activated channel, subfamily M, alpha member 1 (KCNMA1) and Bos taurus integrin, alpha 2b (ITGA2B).

(a) **Protein name**	(b) **Gene name**	(c) **Accession number**	(d) **Ratio**	(e) **p-value**
**Increase in persistently MAP-infected cows.**				
Bos taurus potassium channel with large conductance, calcium-activated, subfamily M, alpha member 1.	*KCNMA1*	ENSBTAP000017697	15.2	0.004
Bos taurus integrin, alpha 2b	*ITGA2B*	ENSBTAP000010741	2.2	0.047
